# Post-extraction maxillary lip entrapment in cats: a prospective study

**DOI:** 10.3389/fvets.2025.1620100

**Published:** 2025-07-09

**Authors:** Robert Marx, Margherita Gracis, Luka Šparaš, Ana Nemec

**Affiliations:** ^1^Animal Hospital Hofheim, IVC Evidensia, Hofheim, Germany; ^2^Clinica Veterinaria San Siro, AniCura, Milan, Italy; ^3^Small Animal Clinic, Veterinary Faculty, University of Ljubljana, Ljubljana, Slovenia

**Keywords:** feline dentistry, maxillary canine tooth extraction, maxillary lip entrapment, buccal bone removal, head morphometrics

## Abstract

**Objective:**

To investigate the prevalence, outcomes, and contributing factors of post-extraction maxillary lip entrapment (MLE) in cats, with a focus on anatomical and surgical technique-related variables.

**Animals:**

Thirty-seven client-owned cats undergoing maxillary canine tooth extraction between December 2022 and November 2024.

**Procedures:**

This prospective study included cats undergoing maxillary canine tooth extraction performed by veterinary dental surgeons across three referral clinics. Specifically, we intended to explore the association between MLE and skull conformation, mandibular canine teeth crown height, distance between the crown tips of the maxillary and mandibular canine teeth, extent of maxillary canine alveolectomy, and presence/absence of caudal teeth on this clinical entity. Post-extraction MLE was classified as mild, moderate, or severe based on clinical findings and treatment requirements.

**Results:**

Post-extraction MLE was observed in 26 cats (70.3%), with 23 of 26 cats (88.5%) being classified as having mild lesions, three (11.5%) with moderate lesions and none with severe lesions. Spontaneous improvement was noted in all mild cases. Only patients with moderate lesions required medical intervention. None of the evaluated factors had any statistically significant impact of the prevalence of MLE.

**Conclusions and clinical relevance:**

Post-extraction MLE in cats is a frequent but predominantly mild and self-limiting complication. Conservative management typically suffices, and advanced imaging in future studies could enhance understanding of predisposing factors and surgical strategies, leading to improved patient outcomes.

## 1 Introduction

Tooth extraction is one of the most commonly performed procedures in veterinary dentistry. While the primary goal of dentistry is to preserve tooth integrity, there are numerous instances where extracting a tooth provides a more favorable outcome for the patient’s overall health (1). Several conditions may warrant this treatment, with stage 4 periodontal disease, advanced tooth resorption with lesions exposed to the oral cavity, and dental trauma with a poor prognosis for tooth-preserving treatments being the most common indications for extraction in cats. Periodontal disease is particularly prevalent, with reported prevalence rates ranging from 13.9 to 96% (2–5). Alveolar bone expansion (ABE), a proposed clinical form of this disease, has been identified in 35% of cats presented for dental evaluation, predominantly affecting the canine teeth (6). This condition often progresses unnoticed, leading to severe inflammation, tooth mobility, and eventual tooth loss in its advanced stages (4).

Tooth resorption affects between 28.5 and 67% of cats and its incidence increases with age (7–9). The maxillary canine teeth were identified in one study as the second most commonly affected teeth after the mandibular molar teeth (10). Moreover, dentoalveolar trauma, particularly involving canine teeth, is common. One study found that the mandibular and maxillary canine teeth were the most frequently injured teeth in dogs and cats presented for oral treatment, accounting for 35.5% of cases, with the overall prevalence of traumatic dental injuries (TDI) reported at 26.2% (11).

Although tooth extraction is considered an effective treatment option, often regarded as the gold standard to alleviate the abovementioned conditions (12), complications can arise in the course of the extraction or during the postoperative period (13). A common complication following the extraction of maxillary canine tooth in cats is maxillary lip entrapment (MLE) with the ipsilateral mandibular canine tooth. Varying degrees of pain and irritation may arise in these cats (14). Several treatment strategies have been proposed to address this complication, including crown height reduction (i.e., odontoplasty followed by application of a bonding agent, or crown amputation and endodontic treatment of the mandibular canine tooth) (15), extraction of the mandibular canine tooth (16, 17) or maxillary canine tooth replacement with an implant (16). Controversy exists regarding the latter method in veterinary medicine (18). Other methods, though unpublished, have been anecdotally reported to show some success, such as reshaping the tip of the mandibular canine tooth with resin composite to reduce its sharpness.

Unfortunately, the lack of sufficient data regarding this condition in cats impedes a comprehensive understanding, leaving guidelines for intervention undefined. Therefore, the present study aims to describe the prevalence and outcome of post-extraction MLE in cats and to provide insights into potential correlations of various anatomical and surgical technique-related factors. Specifically, we intended to explore the influence of skull conformation, mandibular canine teeth crown height, distance between the crown tips of the maxillary and mandibular canine teeth, extent of maxillary canine alveolectomy, and presence/absence of other teeth on this clinical entity.

## 2 Materials and methods

### 2.1 Study population and surgical procedures

Client-owned cats from three veterinary referral clinics undergoing routine dental procedures between December 2022 and November 2024 were included in this study. The procedures were performed by four veterinary dental surgeons, consisting of two AVDC/EVDC (American and European Veterinary Dental Colleges) diplomates (MG, AN) and two EVDC residents (RM, LŠ). All surgeons adhered to the same surgical extraction techniques, allowing reliable comparison of results across patients. To be included in the study, one or both maxillary canine teeth needed to be surgically extracted (19) while the ipsilateral mandibular canine tooth or teeth remained intact. Cats presenting with any malocclusion type, as defined by AVDC classification systems (20), were excluded from the study. No other exclusion criteria were applied. All procedures were performed as clinically indicated and adhered to standard veterinary care protocols; therefore, ethics committee approval was not required, but written informed consent for the procedures was obtained from all clients. Each animal underwent a comprehensive assessment with full-mouth radiography and dental charting, and treatment under general anesthesia including regional nerve blocks for analgesia. Infraorbital nerve blocks using levobupivacaine 0.5% at 0.2 mL per site (21) were performed prior to any extractions all in accordance with procedural guidelines (22). All cats were sent home the same day with analgesia as clinically indicated and detailed discharge instructions and re-check examinations were scheduled within 4 weeks.

### 2.2 Data collection

Detailed demographic data (i.e., age, breed, and sex) were recorded for each patient.

The reasons for extraction of maxillary canine teeth were categorized into three main groups: (1) periodontal disease, (2) tooth resorption, and (3) traumatic dental injuries, with combinations of reasons noted. Furthermore, additional tooth extractions were recorded and the proportion of remaining caudal teeth was evaluated and categorized as either greater than, equal to, or less than 50%.

**Figure 1:**
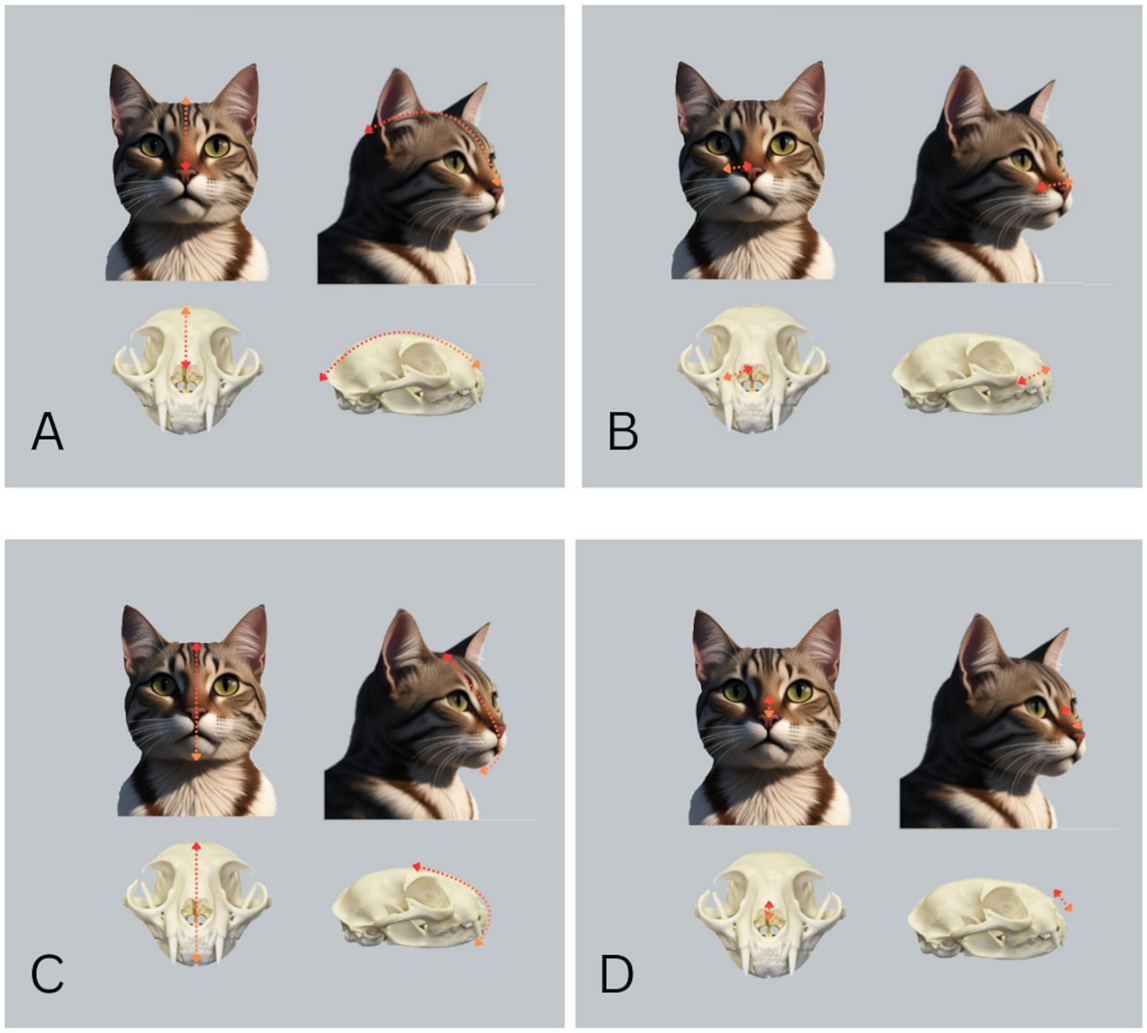
Measurements of craniofacial dimensions in cats. **(A)** Cranial length was measured as the distance from the occipital protuberance to the dorsal tip of the nose (red-orange arrow). **(B)** Muzzle length was determined as the distance from the dorsal tip of the nose to the entrance of the infraorbital canal (red-orange arrow). **(C)** Facial length was measured as a curved line extending from the highest point of the head between the pinnae to the caudal extension of the symphysis assessed by palpation (red-orange arrow). **(D)** The eye-to-nose distance was recorded as the length between the midpoint between the medial canthi of the eyes and the dorsal tip of the nose (red-orange arrow).

To measure and analyze head morphometrics, a modified version of a previously established and published method was utilized (23). Measurements of cranial length, muzzle length, facial length and eye-to-nose distance were conducted by using a calibrated tape measure, with well-defined anatomical landmarks employed to ensure consistency (Figure 1).

The proportional muzzle length ratio was calculated by dividing the muzzle length by the cranial length and expressing it as a percentage by multiplying by 100, while the proportional nose position ratio was determined by dividing the eye-to-nose distance by the facial length and multiplying the result by 100.


$$\begin{array}{l} \text{Proportional Muzzle Length Ratio}\\ =(\text{Muzzle Length}/\text{Cranial Length})\times 100 \end{array}$$

$$\begin{array}{l} \text{Proportional Nose Position Ratio}\\ =(\mathrm{Eye}\;\text{to Nose Distance}/\text{Facial Length})\times 100 \end{array}$$

**Figure 2:**
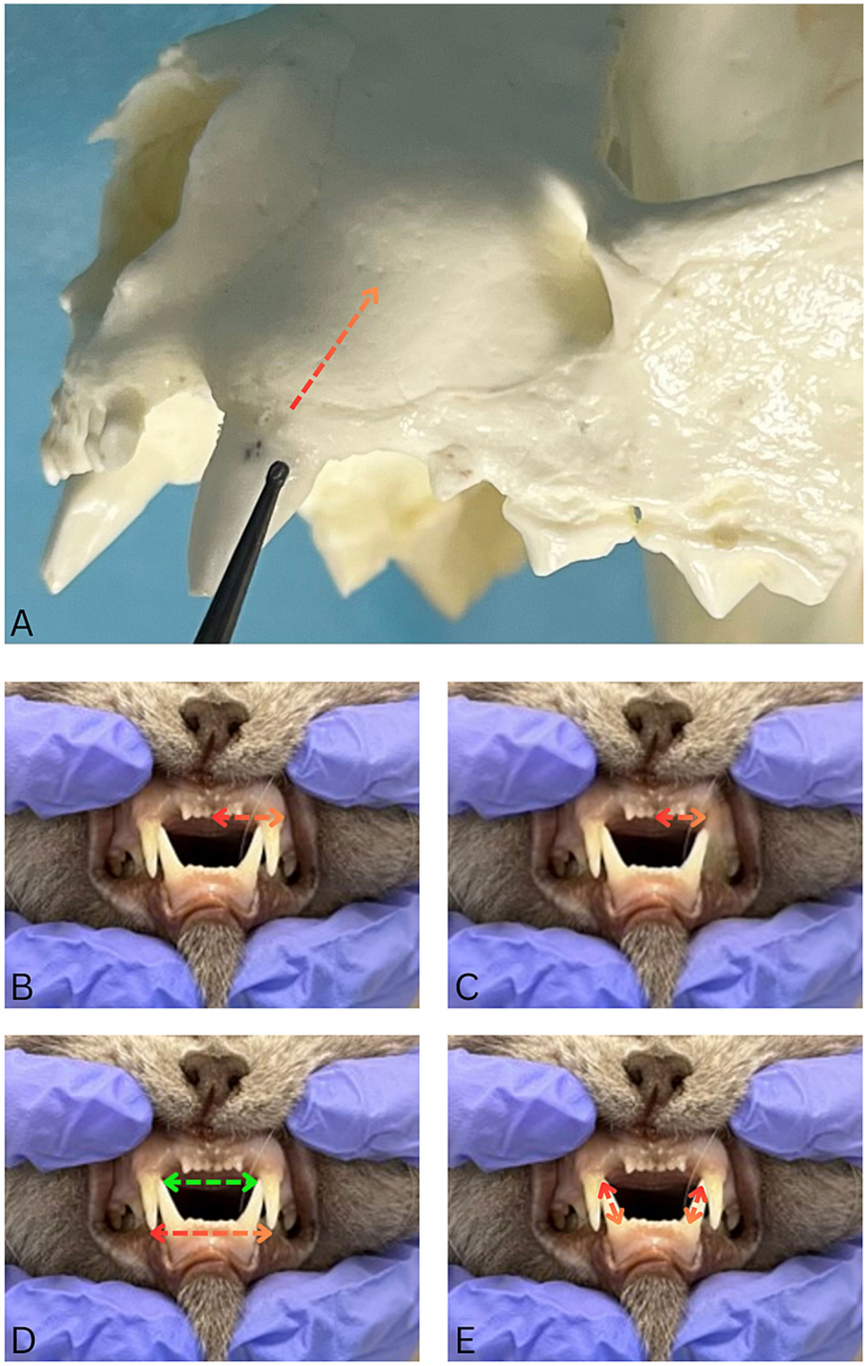
Photographs illustrating dental measurements and bone assessments. **(A)** Apical bone removal was quantified as a percentage of bone removed relative to the tooth root length (red-orange dotted arrow). **(B)** Before-surgery measurement of the distance between the canine tooth jugum and the interincisal midline (red-orange dotted double-arrow). **(C)** After-surgery measurement of the distance between palatal aspect of the vacated alveolus and the interincisal midline (red-orange double-arrow) **(D)**. The distances between the crown tips of right and left maxillary (red-orange double-arrows) and mandibular (green double arrow) canine teeth were recorded. **(E)** The crown height of the mandibular canine teeth was measured clinically from the gingival margin to the tip of the crown (red-orange double-arrows). These teeth were healthy, with no signs of loss of periodontal tissues loss.

To investigate the influence of bone removal during tooth extraction, the extent of alveolar ostectomy was measured in two directions. Apical bone removal was measured from original coronal level of alveolar bone (as noted upon opening mucoperiosteal flap) to the point on the extracted tooth where the bone had been removed. Apical bone removal (i.e., height) (Figure 2A) is given in percentage and categorized into three groups: (1) less than 25%, (2) 25–50%, and (3) more than 50% of the root length. Buccal bone removal (i.e., thickness) was calculated as follows. The distance between the buccal surface of the alveolar bone of the maxillary canine tooth to be extracted and the interincisal midline was measured in millimeters before surgery (Figure 2B). After the surgery the distance between the palatal aspect of the vacated alveolus and the interincisal midline was measured (Figure 2C). The after-surgery measurement was then subtracted from the before-surgery measurement to obtain the amount of buccal bone removed.

The distances between the crown tips of right and left maxillary and mandibular canine teeth were recorded (Figure 2D), along with the crown height of the mandibular canine teeth (Figure 2E). The decision to obtain these data was made shortly after recruiting the first cases, therefore these data are not available for every patient.

**Figure 3:**
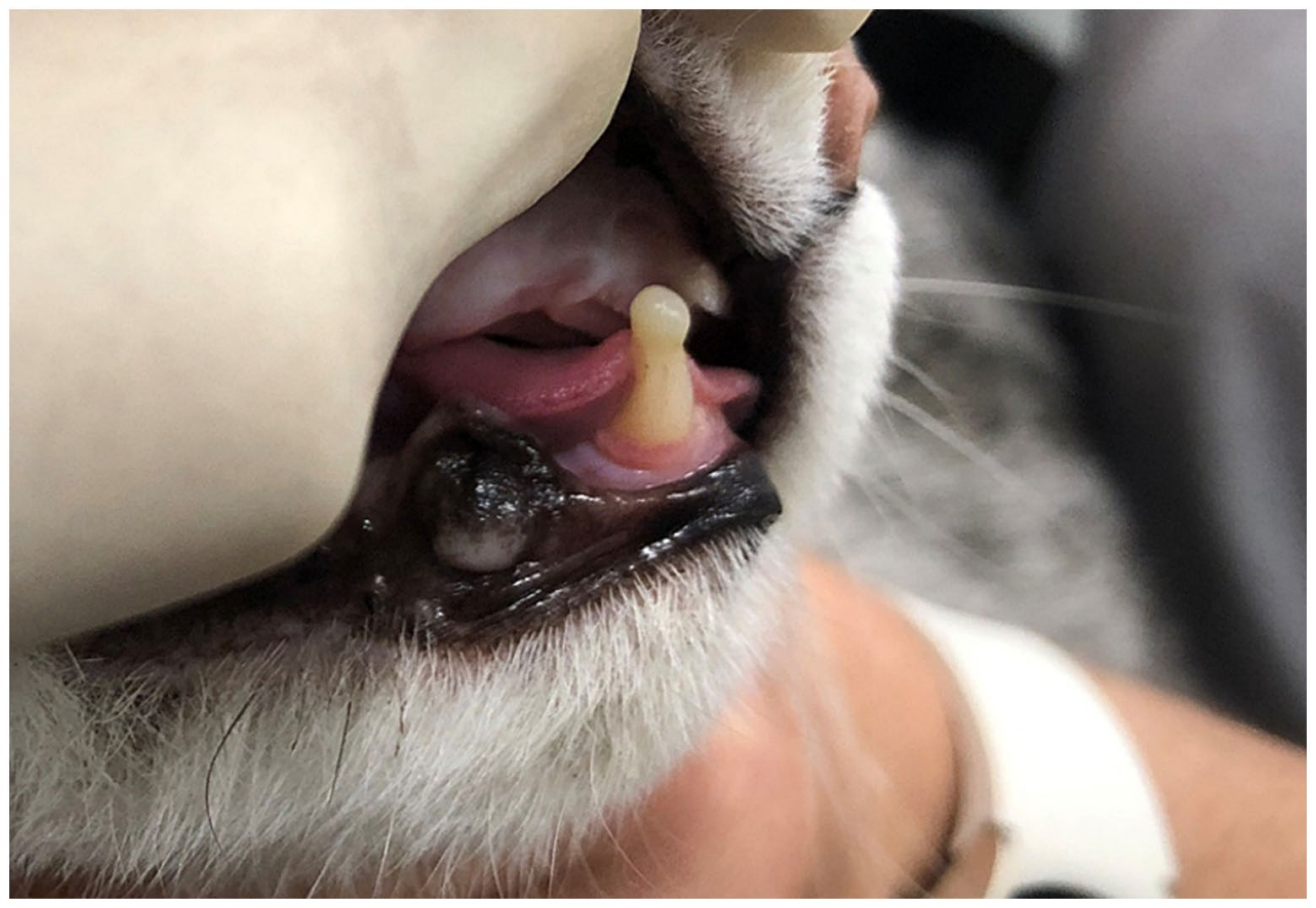
Photograph of a patient 2 months following maxillary canine tooth removal showing composite placement on the ipsilateral mandibular canine tooth.

Additionally, in six of the first 10 cats included in this study (randomly selected using a coin toss) a dental composite material was applied to the tip of the crown of the ipsilateral mandibular canine tooth in the shape of a bullet, based on an unpublished technique. The application was performed as follows: the tooth surface was cleaned and dried, then etched with 37% phosphoric acid for 20 s and rinsed thoroughly. A bonding agent (Bond®, IM3, Duleek, Ireland) was applied and light-cured according to the manufacturer’s instructions. Subsequently, a flowable composite (Silkflow®, IM3, Duleek, Ireland) was shaped into a rounded, bullet-like form at the incisal tip and cured with a standard LED light-curing unit. Final adjustments (polishing) were made to ensure smooth contours and patient comfort (Figure 3).

### 2.3 Follow-up evaluations

For the purpose of this study, MLE was considered to be present if the cat was observed to physically catch its maxillary lip with the mandibular teeth, or if a maxillary lip lesion consistent with trauma from MLE was identified on physical examination. The presence of any MLE immediately post-surgery was noted once the cats had fully recovered and before discharge.

Each cat was then clinically re-checked in person at least once within the first 4 weeks after surgery to monitor immediate postoperative outcomes. Further follow-up assessments were advised at regular intervals, but in some cats follow-up was only conducted if changes or problems were noted, prompting the owners to contact us. These assessments included in-person examinations, as well as remote evaluations through phone calls, emails, and photo submissions provided by the owners. Re-checks were conducted to assess healing of the extraction sites, healing of any previously diagnosed lip lesions, monitor for other complications, and evaluate clinical improvement regardless of presence/absence of physical lip entrapment at the time of re-checks (i.e., the presence of lip lesions was used as evidence that MLE had occurred). The endpoint for follow-up was defined as the resolution of clinical signs, satisfactory healing, and owner-reported improvements in the cat’s behavior and quality of life, with no further treatment required and no signs of discomfort observed.

MLE was classified as mild (MLE was clinically visible without any maxillary lip lesion, or the lesion appeared as a small, well-defined area of hair loss without any signs of inflammation and no treatment was required), moderate (the lesions presented as a localized area of redness, swelling, superficial ulceration and tenderness on the lip and/or extended medical support was given), or severe (the affected area showed pronounced redness, swelling, warmth and deep ulceration/penetrating wound, with signs of pain and discomfort observed when touched, and surgical intervention was necessary to resolve the condition, i.e., crown height reduction with endodontic treatment or extraction of the ipsilateral mandibular canine tooth).

### 2.4 Data analysis

The data were analyzed using R version 4.3.3 (R Core Team, 2024) (24). We assessed the normality of the data using the Shapiro–Wilk test and visual inspection of Q-Q plots. When normality was met, independent samples t-tests were performed, reporting means and standard deviations, with Cohen’s d as the effect size. For non-normally distributed variables, Mann–Whitney U tests were conducted, with medians and interquartile ranges reported, and rank biserial correlation as the effect size. Categorical data were analyzed using Fisher’s Exact Test, with Cramér’s V used to assess the effect size. *p*-values were considered significant at the 0.05 level. Morphometric variables (nose position ratio, muzzle length ratio, maxillary canine crown tip distance, mandibular canine crown tip distance, crown height of the mandibular canine teeth) and the presence of remaining caudal teeth were analyzed at the cat level, whereas surgical variables (amount of buccal alveolar bone removal and the extent of alveolar ostectomy in the apical direction) were analyzed at the tooth level. Cat-level analysis involved averaging values per individual in cats with multiple extractions, while tooth-level analysis treated each tooth as a separate data point.

## 3 Results

### 3.1 Study population

This study included 37 cats, consisting of 14 neutered males, seven intact males, 11 neutered females, and five intact females. The cats represented seven breeds: European Shorthair (*n* = 27), British Shorthair (*n* = 2), Maine Coon (*n* = 3), Ragdoll (*n* = 1), Sacred cat of Burma (*n* = 1), Mix (*n* = 2), and Siberian cat (*n* = 1). The mean age of the cats was 8.03 years (SD = 3.87), and it ranged from two to 17 years. There was no significant difference in age between groups [*t* (35) = −0.21, *p* = 0.834]. Similarly, no significant difference in sex distribution between groups was found (Fisher’s Exact Test, *p* = 0.442). These results suggest that neither age nor sex are likely to act as confounding variables in the analysis.

Periodontal disease (stage 3 or 4) was the most common reason for tooth extraction, reported in 21 cats (56.8%), followed by traumatic dental injuries in eight cats (21.6%), tooth resorption in four cats (10.8%), and a combination of reasons in four cats (10.8%). In nine cats both maxillary canine teeth were extracted and 28 cats had only one maxillary canine tooth extracted (17 left and 11 right).

### 3.2 Prevalence of MLE and follow-up

Eleven cats (29.7%) did not develop MLE during the study period.

Immediately after surgery, MLE was observed in 13 cats (35.1%), all of which were classified as mild. Of these, nine cats involved the removal of one maxillary canine tooth, and four involved the extraction of both maxillary canine teeth. In the subgroup of four cats where both maxillary canine teeth were removed, one showed no signs of MLE during the first clinical recheck 2 weeks later, along with three other cats from the subgroup of nine with only one tooth removed.

**Figure 4:**
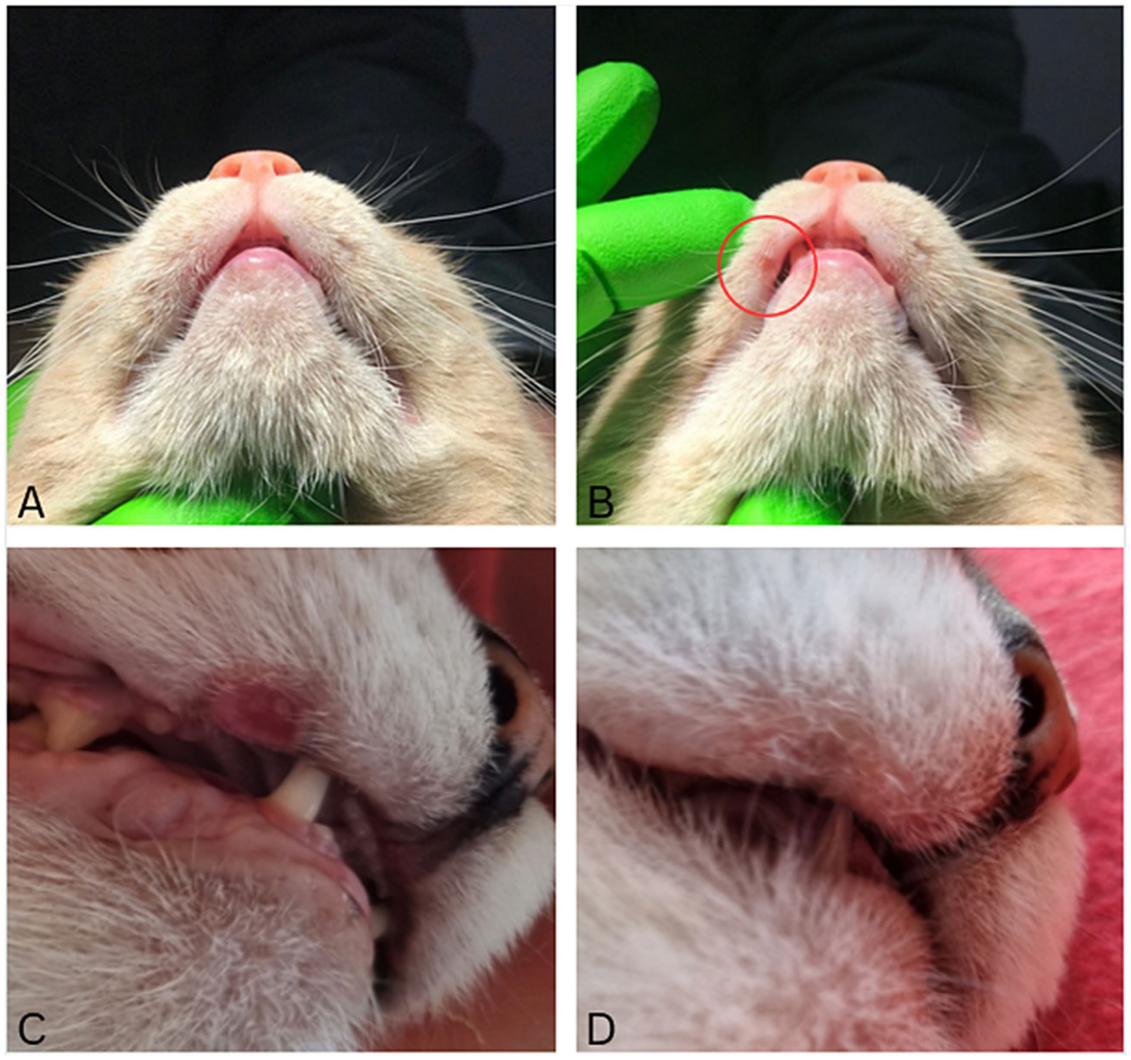
Clinical photos illustrating lesion severity. **(A)** Lip lesions can be easily missed without a thorough clinical examination. **(B)** Same animal as in **A**. Mild lesions appeared as small, well-defined areas of hair loss (red circle), requiring no treatment. **(C)** Moderate lesion presented as localized areas of redness, swelling and ulceration. **(D)** Same animal as in **C** 2 months later, demonstrating significant improvement of the lip lesion after no additional therapy.

At the first clinical recheck, conducted within the first 4 weeks after surgery, MLE was detected in a total of 26 cats (70.3%). Among these, 23 cats (88.5%) were classified as having mild lesions (Figures 4A,B), while three cats (11.5%) presented with moderate lesions (Figures 4C,D). For two cats with moderate lesions extended medical support was provided, including the administration of a nonsteroidal anti-inflammatory drug (meloxicam, starting dose 0.1 mg/kg, SID, po, Metacam®, Boehringer Ingelheim, Ingelheim, Germany) and/or the application of a topical anti-inflammatory and protective mucosal agent twice daily (Oraflogo gel, Medicinalis GmbH, Klagenfurt, Austria) for up to 7 days to promote mucosal healing and alleviate discomfort. None of the cats in this study showed severe lesions requiring surgical intervention.

Eight of the 26 cats (30.8%) with MLE had bilateral maxillary canine teeth extractions, but only four had lesions bilaterally.

Of the 26 cats with MLE noted, 24 were rechecked at least twice. The overall longest follow-up period was 14 months, while the shortest was 6 weeks. In all these cases, at the last re-check improvement was observed, including a reduction in lesion size, visible signs of healing, decreased inflammation, and a lower frequency of lip catching (Figures 4C,D).

The 11 cats that did not show MLE at the first clinical recheck (including four cats that had immediate entrapment after surgery) were not followed up further.

### 3.3 Head morphometry, dental anatomy and MLE

In cats with MLE the proportional nose position ratio had a mean of 21.64% (SD = 3.02%), and in cats without MLE it had a mean of 21.43% (SD = 3.36%). An independent samples t-test revealed no significant difference between the groups for the proportional nose position ratio [*t* (35) = −0.19, *p* = 0.851]. The effect size, Cohen’s d, was small (*d* = −0.07), with a 95% confidence interval ranging from −0.77 to 0.64.

The proportional muzzle length ratio in cats with MLE had a median of 23.12% (IQR = 7.09%), and in cats without MLE it had a median of 20.53% (IQR = 6.45%). A Mann–Whitney U test indicated no significant difference between the groups for the proportional muzzle length ratio (*U* = 99.0, *p* = 0.148). The effect size, rank biserial correlation, was moderate (*r* = 0.31).

The maxillary canine teeth crown tip distance was measured in 22 cats with MLE, yielding a median of 19.00 mm (IQR = 3.00 mm), and in seven cats without MLE, yielding the same median of 19.00 mm (IQR = 1.00 mm). A Mann–Whitney U test showed no significant difference between the groups for the maxillary crown tip distance (*U* = 69.50, *p* = 0.717), with a small (*r* = 0.10) effect size.

Mandibular canine teeth crown tip distance was analyzed in 22 cats with MLE showing a mean of 16.23 mm (SD = 2.37 mm), and in seven cats without MLE, with a mean of 15.71 mm (SD = 1.70 mm). Results of the independent t-test revealed no significant difference in mandibular canine crown tip distance between the groups [*t* (25) = − 0.53, *p* = 0.602]. The effect size was small [*d* = −0.23, 95%CI (−1.08, 0.64)].

Crown height of the mandibular canine teeth was evaluated in 26 cats with MLE, showing a median of 8.00 mm (IQR = 2.75 mm), and in 11 cats without MLE, which had a median of 8.00 mm (IQR = 1.00 mm). When comparing the crown height of the mandibular canine teeth, no significant difference was found between the groups (*U* = 127.50, *p* = 0.608), as indicated by the Mann–Whitney test. The rank biserial correlation effect size was small (*r* = 0.11).

### 3.4 Surgical procedures and MLE

The amount of buccal alveolar bone removal (thickness) was measured in 26 teeth from 20 cats with MLE, with a median of 4.0 mm (IQR = 1.5 mm), compared to seven teeth in six cats without MLE, where the median was also 4.0 mm (IQR = 2.0 mm). The results of the Mann–Whitney test for the amount of buccal alveolar bone removal showed no significant difference between the groups (*U* = 79.50, *p* = 0.621), with a small effect size [*r* = 0.13; CI (−0.55, 0.35)].

When the extent of alveolar ostectomy in apical direction was evaluated at the tooth level, for teeth with MLE the extent was <25% in 4 teeth (11.8%), 25–50% in 19 teeth (55.9%), and >50% in 11 teeth (32.4%). For teeth without MLE the extent was <25% in none, 25–50% in eight teeth (66.7%), and >50% in four teeth (33.3%). To assess the association between MLE and the extent of alveolar ostectomy at the tooth level, a Fisher’s Exact Test was performed. The analysis showed no significant difference between groups (*p* = 0.680). The corresponding effect size, expressed as Cramér’s V, was 0.19 [95% CI (0.11, 0.39)], suggesting a small level of association.

Before this intervention was aborted, permanent composite placement was performed at the time of dental extraction in three (11.5%) of the cats that at first clinical re-check showed MLE (two mild and one moderate) and in three (27.3%) of the cats that did not develop MLE.

### 3.5 Remaining caudal teeth

Of the 26 cats with MLE, 18 (69.2%) had more than 50% of their caudal teeth remaining, while 8 (30.8%) had less than 50% of caudal teeth remaining. Of the 11 cats without MLE 10 (90.9%) had more than 50% of their caudal teeth remaining, and 1 (9.1%) had less than 50% remaining. Fisher’s Exact Test revealed no significant difference between the groups (*p* = 0.229). The strength of the association was 0.23, indicating a small to moderate effect size, with a 95% confidence interval of 0.09 to 0.37.

## 4 Discussion

Preserving the integrity of canine teeth is a primary focus in veterinary dental care, as they play a crucial role in overall oral health and function (26, 27). When extraction of a canine tooth becomes necessary, it is vital for veterinary dentists to prioritize strategies that minimize complications.

To the best of our knowledge, this is the first study to investigate the prevalence and outcomes of post-extraction MLE in cats, with a focus on identifying potential correlations with anatomical and surgical factors.

This study included 37 client-owned cats that underwent unilateral or bilateral maxillary canine teeth extractions with remaining ipsilateral mandibular canine tooth/teeth. Immediately post-surgery, mild MLE was observed in only 13 cats, but interestingly the number of affected individuals increased at the first clinical recheck, when MLE was observed in 70.3% (*n* = 26) of cats. Bone and soft tissue remodeling during the healing process of an extraction wound in cats is expected within a few weeks (25), and it may be the reason why post-extraction MLE was observed more frequently at the recheck visit.

The majority of cats with MLE (88.5%, *n* = 23) exhibited mild lesions that did not require treatment. Also, among the three cases classified as moderate, only two required extended medical intervention. No cats with severe entrapment were recorded, therefore surgical intervention was not required in any patient.

In all cats with MLE-related lesions, improvement was observed with only a minority of cats requiring medical support. On a longer term, MLE is predominantly self-limiting, with minimal need for intensive therapeutic measures in most instances. Similarly, previous research on ferrets has documented post-extraction MLE occurring in 44.4% of cats with a self-limiting nature of the entity (28).

Cephalometric parameters and facial indices were investigated as contributing factors to the development of post-extraction MLE. The proportional muzzle length ratio was slightly bigger in cats with MLE, with a moderate effect size. This could indicate that a longer muzzle relative to cranial length increases the risk of MLE. However, due to the lack of statistical significance, it is not appropriate to draw a definitive conclusion at this point.

We also analyzed the influence of specific dental anatomy that could contribute to the development of MLE. Both groups had similar measurements in mandibular canine teeth crown height, distance between the maxillary canine teeth crown tips and distance between the mandibular canine teeth crown tips, indicating that there is probably no influence of these factors on MLE.

Anecdotally, the amount of bone removal during maxillary canine tooth extraction has been implicated in the development of post-extraction MLE. However, patients in our study had a similar median amount of buccal bone removal of 4.0 mm in thickness and similar extent of alveolar ostectomy in apical direction regardless of the development of MLE. Therefore, the amount of bone removal in either direction does not seem to play an important role in the development of MLE. In ferrets MLE was observed in a high proportion of cases although no bone was removed in any direction in any of the patients, as maxillary canine teeth were removed in a closed manner (28). Similar to ferrets, in the authors experience MLE may be observed in cats even before removal of the maxillary canine tooth, particularly if the crown of the tooth is very short due to fracture. Therefore, in cats with alveolar bone expansion, buccal bone should be removed as needed as a mean of debridement for focal osteomyelitis (29) and to allow for tension-free closure of the extraction site without likely impacting the development of MLE.

This study also explored the potential role that further extractions may have played in the development of MLE, because of a hypothetical increase in bite depth. However, 69.2% of cats retained more than half of their caudal teeth. Therefore, the influence of changes of the caudal dentition on the development of MLE appears to be minimal. On the other hand, even the extraction of the canine teeth could influence the bite depth, but to evaluate this hypothesis more precise cephalometric studies, possibly utilizing advanced diagnostic imaging modalities (i.e., CT), would be required.

The placement of a dental composite on the mandibular canine tooth has not been formally reported in the literature, but is anecdotally utilized as a means of preventing MLE in cats. In this study, we aborted using this intervention early based on the authors’ observation that this method was ineffective. This approach remains an intriguing concept that warrants further investigation to optimize placement techniques and evaluate its impact on clinical outcomes.

The study’s limitations highlight areas for future improvement. The relatively small sample size precluded robust statistical analysis. Many estimates had wide confidence intervals, indicating that the study may have been underpowered to detect effects of small and modest size. As illustrated by the post-hoc power analysis for proportional muzzle length, as one example, larger samples would help clarify whether apparent trends reflect true group differences. To detect an effect size equivalent to the rank biserial correlation of 0.31 (converted to Cohen’s d ≈ 0.65), approximately 38 participants would be needed per group (about 76 total) to achieve 80% power at an alpha level of 0.05 using a two-sided test. In addition to the sample size limitation, different surgeons may have introduced potential errors. Future research should incorporate advanced 3D imaging technologies, such as computed tomography (CT) or cone-beam CT (CBCT) to enhance the precision and accuracy of measurements, providing deeper insights into the cephalometric variations (30, 31) that may predispose cats to post-extraction maxillary lip entrapment. Such advancements could refine surgical techniques and contribute to improved feline welfare.

## 5 Conclusion

Despite the high prevalence of post-extraction MLE in cats, the complications were predominantly mild, with improvement over time in all cats seen in this study. These findings suggest that a conservative approach may be appropriate when managing this condition.

Future research utilizing advanced imaging techniques such as CT or CBCT, alongside anesthetized reexaminations, could provide deeper insights into the role of head and dental arch shape as well as the role of surgical interventions, leading to improved management strategies.

## Data Availability

The original contributions presented in the study are included in the article/supplementary material, further inquiries can be directed to the corresponding author/s.
